# Cemented versus uncemented fixation of femoral components in 2-stage hip revision arthroplasty to treat periprosthetic joint infection: a cohort study on 94 patients comparing the risks for relapse and reoperation

**DOI:** 10.2340/17453674.2025.44923

**Published:** 2025-12-11

**Authors:** Georgios PALECHOROS, Anders BRÜGGEMANN, Nils P HAILER

**Affiliations:** Department of Surgical Sciences/Orthopaedics & Hand Surgery, Uppsala University, Uppsala, Sweden

## Abstract

**Background and purpose:**

Both cemented and uncemented stem fixation is used in 2-stage hip revision arthroplasty addressing periprosthetic joint infection (PJI). We aimed to compare the risk of infection relapse and the risk of reoperation for any reason between uncemented and cemented stem fixation.

**Methods:**

Patients who underwent 2-stage hip revision arthroplasty for PJI between 2005 and 2020 were included. Data on baseline demographics, implant type, and microbiological and antibiotic treatment data was obtained from a local registry and medical records. Kaplan–Meier analysis compared relapse-free survival and reoperation-free survival between uncemented (n = 60) and cemented (n = 34) stems. Cox regression models were fitted to assess adjusted hazard ratios (aHR) for the risk of relapse or reoperation with 95% confidence intervals (CIs).

**Results:**

94 patients underwent 2-stage revision hip arthroplasty for PJI. Unadjusted 2-year relapse-free survival rates were 95% (CI 89–100) for patients with uncemented stem fixation and 97% (CI 90–100) for those with cemented fixation. Reoperation-free survival at 10 years was 82% (CI 70–95) for patients with uncemented fixation and 61% (CI 43–85) for those with cemented fixation. Using cemented fixation as the reference, the aHR for infection relapse was 2.0 (CI 0.2–20.1, P = 0.6) for uncemented fixation, whereas the aHR for reoperation was 0.3 (CI 0.1–0.9, P = 0.03).

**Conclusion:**

We showed no statistical difference in the risk of infection relapse, but uncemented stem fixation in 2-stage revision arthroplasty for PJI was associated with a reduced risk of reoperation for any reason. Uncemented stems may thus be a suitable choice in 2-stage revisions for PJI when this concept is believed to provide better fixation.

2-stage hip revision arthroplasty in prosthetic joint infection (PJI) involves the removal of the infected implants and substitution with an articulating or a non-articulating spacer containing antibiotic-loaded bone cement (ALBC), followed by a course of antibiotic therapy. After this period, the spacer is replaced by new implants [1] using cemented or uncemented fixation. In contrast, 1-stage revision arthroplasty involves exchanging the prosthesis in a single surgical procedure [2], with proposedly similar results to 2-stage procedures [3]. 2-stage revision arthroplasty remains a widely used method in PJI cases with difficult-to-treat bacteria, polymicrobial PJI, in cases with severely compromised soft tissue, or after failed DAIR or 1-stage revision [4–6].

ALBC has been shown to reduce the risk of PJI after primary arthroplasty [7]. Therefore, using ALBC has in some countries been widely regarded as the gold standard for 1- and 2-stage revisions treating PJI [8]. However, conclusive evidence is lacking to support the assertion that ALBC reduces the risk of infection relapse after 2-stage revision arthroplasty.

We investigated whether the concept of uncemented stem fixation in 2-stage revision arthroplasty is associated with inferior outcomes in terms of infection (relapse or new PJI) or reoperation.

## Methods

### Study design

This is a comparative retrospective cohort study reported according to the STROBE guideline.

### Population

Our cohort comprised patients who underwent 2-stage revision hip arthroplasty for PJI at Uppsala University Hospital between 2005 and 2020. The starting year was determined by the introduction of digital medical records at Uppsala University Hospital. A minimum 2-year follow-up period was implemented for all patients; those operated on after 2020 were therefore excluded. No additional exclusion criteria were applied.

### Surgical procedures

According to our local treatment guidelines, factors such as the presence of difficult-to-treat pathogens, polymicrobial PJI, culture-negative PJI, compromised soft tissue, and failed previous DAIR treatment weighed in favor of 2-stage instead of 1-stage revision surgery. During the study period the threshold for choosing 1- instead of 2-stage revision procedures in the treatment of PJI was gradually lowered, following international trends, but this trend was similarly observed in both the exposed and the control group [9].

All surgical procedures were conducted with patients in the lateral decubitus position through either a direct lateral approach or an extended trochanteric osteotomy (ETO). Stage 1 of the procedure involved the removal of infected implants and all foreign material (sutures, bone cement, cement restrictor, cerclage wires), followed by the collection of no less than 5 tissue samples for microbiological analysis. Tissue debridement was performed, and the surgical field was irrigated with at least 6 L of saline solution. Following debridement and irrigation, all surgical cavities, including the acetabulum and femur, were filled with antiseptic-soaked (betaine/polyhexanide; Prontosan, B. Braun Medical, Melsungen, Germany) surgical gauzes, after which the wound was provisionally closed. The antiseptic was left to act for 15–20 minutes, during which time the surgical field was re-draped, and the surgical staff changed gowns, gloves, and helmets. Finally, gauzes were removed, the field was irrigated again, and a spacer made of, or covered by, ALBC was inserted using new instruments.

During stage 2, the spacer was removed and new implants were inserted. Tissue samples were again sent for cultures at stage 2 to confirm treatment success. For cemented stems, endofemoral pressure was optimized using a modern cementing technique that included a distal cement restrictor, irrigation immediately preceding retrograde cement application, and femoral canal tamponade. For uncemented stems, conical straight stems (modular or not) were used for diaphyseal press-fit fixation (see Table 2). Cerclage wires were inserted following the use of an ETO. In patients operated on through an ETO, uncemented modular stems with diaphyseal fixation were used if bone stock was supportive. In cases of deficient bone stock, cemented modular stems were used. Patients in whom the revised stem was removed endofemorally during stage 1 mainly received uncemented stems with diaphyseal fixation (modular or monoblock) during stage 2, unless non-supportive diaphyseal bone dictated otherwise.

### Exposure and outcomes

The second stage of the 2-stage revision arthroplasty was defined as the index surgery. For patients treated with more than 1 x 2-stage revision, index surgery was defined as stage 2 of the chronologically first 2-stage revision. Depending on stem fixation, the patients were divided into 2 groups. We defined patients treated with uncemented stem fixation at index surgery as the exposed group, and patients treated with cemented stem fixation as unexposed controls. Our primary outcome was survival free from infection relapse within 2 years after index surgery.

We defined infection relapse as persistent or recurrent infection with the same pathogen as the one found during stage 1 foregoing the index surgery, identified in the same joint, within 2 years from index surgery. As secondary outcomes, we defined survival free from reoperation, i.e., further revision arthroplasty or other major surgery for any reason, i.e., loosening, periprosthetic fracture, recurrent dislocation, or PJI (relapse or new), within 10 years, survival free from reoperation for aseptic reasons, and survival free from both PJI and reoperation, also within 10 years. This latter compound outcome includes relapse of PJI caused by the same pathogen, and development of secondary PJI caused by a new microorganism, even if not surgically treated.

### Data collection

The occurrence of relapse and reoperation for any reason was identified in all patients by reviewing medical charts. The statistical analysis for the outcomes of infection relapse and reoperation was performed on the entire cohort of 94 patients. We verified that the indications, diagnostics (prior and following the index surgery) and antibiotic treatment were correct for all patients in the study cohort. The European Bone and Joint Infection Society (EBJIS) criteria [10] were applied retrospectively for the culture-negative cases to verify that the indication for surgery or other treatment was correct.

### Pathogens

Synovial fluid was collected prior to the 2-stage revisions. In cases of dry tap or negative synovial fluid cultures, tissue samples were obtained surgically prior to the 2-stage revision. Tissue samples were also collected during stage 1 of the 2-stage revision arthroplasty. During and after a 10–14-day incubation period at Uppsala University Hospital microbiology laboratory, the culture plates were examined for bacterial growth and tested for antibiotic resistance (antibiogram/resistogram). When available, tissue samples and synovial fluid were also sent for 16S rRNA gene sequencing. In collaboration with the hospital’s department for infectious diseases we analyzed the findings to define the relevant pathogen(s) for each PJI patient. Polymicrobial PJIs, PJIs caused by pathogens that were resistant to antibiotics active against biofilm, PJIs in which no suitable antibiotics with activity against biofilm were available, PJIs caused by cutibacteria, and culture-negative PJIs were considered “difficult-to-treat” [11].

### Statistics

Relapse-free and reoperation-free survival were estimated using the Kaplan–Meier method. Adjusted hazard ratios (aHR) for our primary and secondary outcomes, with 95% confidence intervals (CI), were estimated using Cox regression models adjusted for age, as a continuous covariate, sex, and the presence of difficult-to-treat microorganisms. The proportional hazards assumption was evaluated by plotting and evaluating Schoenfeld’s residuals (using R version 4.3.1; R Foundation for Statistical Computing, Vienna, Austria), and no violations were observed.

### Ethics, data sharing plan, funding, use of AI, and disclosures

This study was conducted following the guidelines of the Helsinki Declaration and approval for this study was granted by the Swedish Ethical Review Authority (entry number 2016/214, date of issue June 14, 2016; and entry number 2022-03466-02, date of issue October 14, 2022). Individual patient consent was not obtained because, according to Swedish law and the decision by the Ethical Review Authority, this is not a requirement in an observational study of this design. According to the Swedish Ethical Review Authority and the Swedish Patient Data law, nothing other than aggregated data can be shared. Following application to the Ethical Review Authority access to the dataset may be granted. Funding was provided by Region Uppsala (under the agreement between the Swedish government and the county councils, “ALF”), Skobranschens Utvecklingsfond, and the Swedish Research Council (N.P.H.: 2021-00980). AI tools were not used. Complete disclosure of interest forms according to ICMJE are available on the article page, doi: 10.2340/17453674.2025.44923

## Results

**Figure 1:**
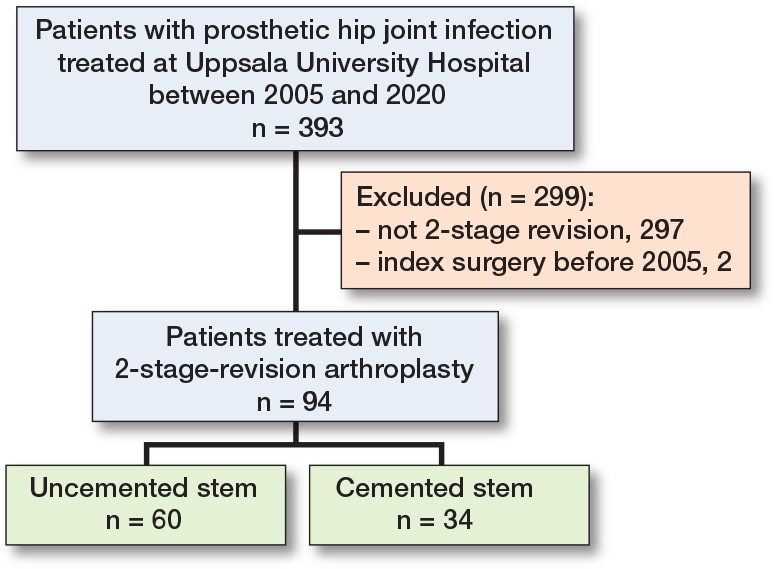
Study flowchart.

We identified 393 incident cases of PJI of the hip in our local PJI registry. Of these, 297 were treated with DAIR or 1-stage revision arthroplasty; surgical intervention for 2 patients occurred before 2005 and they were excluded as we had no access to digital records. Thus, the final cohort consisted of 94 patients (Figure 1).

### Characteristics of the study population

The median age at index surgery was higher and the follow-up time shorter in the group of patients with cemented stem fixation (Table 1). Each group comprised an equal proportion of male and female participants. The follow-up time for the entire cohort was median 6.7 (interquartile range [IQR] 3.4–9.7) years. Most patients in both groups had previously undergone primary THA, with DAIR being the second most common procedure preceding the index surgery. A variety of stems were used at index surgery. The Lubinus SPII (Waldemar Link GmbH, Hamburg, Germany) was the most common stem in the group of patients with cemented stems. Most uncemented stems were Revitan (Zimmer Biomet Inc, Warsaw, IN, USA) and Wagner SL Revision (Zimmer Biomet Inc). In 2 cases, stems designed for uncemented fixation (1 Wagner, 1 Revitan) were cemented, once due to the lack of an implant of appropriate diameter, and once due to poor bone stock (Paprosky IV) that did not allow for stable uncemented fixation. None of these patients were revised. Most patients in both cohorts received the Avantage dual mobility cup (Zimmer Biomet Inc) on the acetabular side. ALBC served as the method of cup fixation in combination with 43 (72%) uncemented stems and 33 (97%) cemented stems (Table 2).

**Table 1:** Characteristics of the study population, demographics, implant-related history, and surgical methods

Item	Cemented stem n = 34	Uncemented stem n = 60
Sex (female / male)	10 / 24	20 / 40
Age at index surgery ^**a**^	72 [68–78]	68 [58–74]
Follow-up time, years ^**a**^	4.2 [3.0–7.6]	7.8 [5.2–10]
Last surgery before stage 1		
Primary arthroplasty	21	28
DAIR	9	17
Stem revision	2	6
Cup revision	1	6
Total revision	1	3
Use of ETO	3	25
Articulated spacer (yes / no)	30 / 4	54 / 6
Type of prosthesis revised at stage 1 ^b^ (primary / secondary)	27 / 7	41 / 19
Years to index surgery since last surgery (stage 1 excluded) ^**a**^	1.3 [0.6–3.8]	1.7 [0.8–3.3]

a Values are median [interquartile range]

b Prior to index surgery

DAIR = debridement antibiotics implant retention;

ETO = extended trochanteric osteotomy.

**Table 2:** Implants inserted during index surgery (defined as the second stage of the 2-stage revision arthroplasty). Values are count

Item	Cemented stem n = 34	Uncemented stem n = 60
Stem model		
Revitan	1	28
Wagner SL Revision	1	26
Wagner Cone	0	4
Restoration	0	2
Lubinus	31	0
MP-Link	1	0
Type of ALBC for femoral stem		
COPAL G+V	18	–
Palacos R+G + manually mixed vancomycin	6	–
Palacos R+G	4	–
COPAL G+C	2	–
No information	4	–
Cup model		
TM-revision shell + Avantage	6	22
Avantage	23	20
Trilogy	0	14
Trilogy + cemented Avantage	1	0
Avantage Reload	0	2
Continuum	0	2
Lubinus	2	0
Müller+Avantage	1	0
Pinnacle	1	0
Fixation method of the bone–cup interface		
Uncemented	8	39
Cemented	26	21

ALBC = antibiotic-loaded bone cement;

COPAL G+V = ALBC containing 0.5 g gentamicin and 2 g vancomycin per 40 mL (Heraeus Medical GmbH);

Palacos R+G = ALBC containing 0.5 g gentamicin per 40 mL (Heraeus Medicall GmbH);

COPAL G+C = ALBC containing 1 g gentamicin and 1 g clindamycin per 40 mL, (Heraeus Medical GmbH).

The most common pathogens in both groups were coagulase-negative staphylococci (CoNS). Staphylococci species, including *S. aureus* and CoNS, accounted for over half of the cases in both groups. Cases of PJI with difficult-to-treat bacteria as defined earlier were distributed evenly between the 2 groups (Table 3).

**Table 3:** Microbiology of the organisms identified in the treated joint prior to index surgery (relevant pathogens for the PJI treated). Values are count

Pathogen	Cemented stem		Uncemented stem	
	All (n = 34)	Difficult-to-treat (n = 18)	All (n = 60)	Difficult-to-treat (n = 29)
CoNS	13	6	29	11
Cutibacterium	3	3	9	9
Streptococci	4	1	6	1
*Staphylococcus aureus*	6	1	5	1
Polymicrobial	4	4	3	3
Negative cultures	2	2	2	2
*Enterococcus faecalis*	0	0	2	2
*Enterobacter cloaca*e	0	0	1	0
Haemophilus	0	0	1	0
Pseudomonas	0	0	1	0
*Clostridium perfrigens*	0	0	1	0
*Escherichia coli*	1	1	0	0
Finegoldia	1	0	0	0

CoNS = Coagulase-negative staphylococci.

Spacer complications were rare, with 1 patient in each group reoperated on with spacer exchange due to spacer dislocation. PJI was treated successfully in both patients.

### Relapse and reoperation

**Figure 2:**
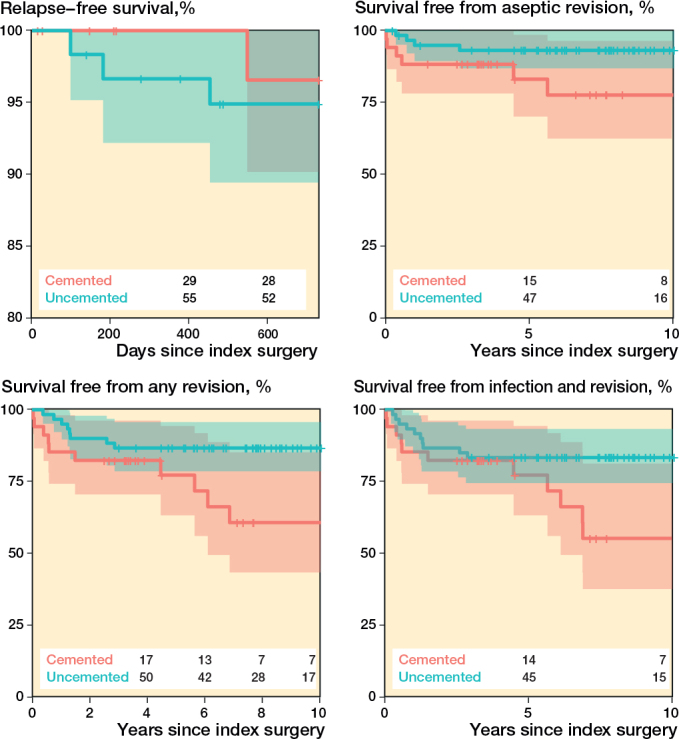
Kaplan–Meier curves, illustrating survival free from relapse (upper left), survival free from reoperation for reason other than infection (upper right), survival free from any reoperation (lower left) and survival from both reoperation and infection, relapse, or new (lower right). The shaded areas indicate 95% confidence intervals and numbers are cases at risk at the corresponding time point.

4 patients experienced infection relapse within 2 years: 3 of 60 patients with uncemented stems and 1 of 34 with cemented stems. The unadjusted relapse-free survival after 2 years was thus 95% (CI 89–100) for patients with uncemented stem fixation and 97% (CI 90–100) for those with cemented stem fixation (Figure 2). The adjusted hazard ratio (aHR) for infection relapse was 2 (CI 0.2–20, P = 0.6) for patients with uncemented stem fixation compared with patients with cemented stem fixation as the reference.

After the index surgery, 18 patients required further surgical intervention, 8 in the uncemented stem group and 10 in the cemented stem group (Table 4 and 5). The reoperation-free survival after 10 years was 82% (CI 70–95) for patients with uncemented stem fixation and 61% (CI 43–85) for those with cemented stem fixation (see Figure 2). Relative to the cemented group, the aHR for reoperation in the uncemented group, irrespective of cause, was 0.3 (CI 0.1–0.9, P = 0.03), while the aHR for aseptic reoperation, i.e., excluding infection as the underlying diagnosis, was 0.3 (CI 0.1–1.1, P = 0.06; Figure 2). For the compound outcome of survival free from both infection and reoperation, the aHR was 0.4 (CI 0.2–1.0, P = 0.06; Figure 2).

**Table 4:** Outcomes

Outcome	Cemented stem		Uncemented stem	
	All (n = 34)	Reoperated (n = 10)	All (n = 60)	Reoperated (n = 8)
PJI relapse	1	1	3	1
PJI successfully treated	33	–	57	–
Implant loosening	3	3	3	3
New PJI	4	3	3	3
Dislocation	2	2	1	1
Periprosthetic fracture	1	1		
Reoperated for any reason within 10 years	–	10	–	8
Survival free from both PJI and reoperation after 10 years	23	–	50	–

PJI = periprosthetic joint infection.

**Table 5:** Patients with relapse after index surgery: microbiology, antibiotic-loaded bone cement presence, and treatment

Type of prosthesis before index surgery	Pathogen at index surgery	Difficult-to-treat	Cement on cup site at index surgery	Treatment
Uncemented stem				
1 Secondary	CoNS	No	Yes (G+V)	2-stage re-revision
2 Primary	CoNS	No	No	Antibiotic suppression
3 Secondary	*S. aureus*	Yes	No	Antibiotic suppression
Cemented stem				
1 Primary	Streptococci	No	Yes (G+V)	2-stage re-revision

CoNS = coagulase-negative staphylococci; G+V, see Table 2.

## Discussion

We aimed to compare the risk of infection relapse and the risk of reoperation for any reason between uncemented and cemented hip stem fixation in 2-stage revision of hip PJI. We showed no statistical difference in risk of infection relapse but uncemented stem fixation in 2-stage revision arthroplasty for PJI was associated with a reduced risk of reoperation for any reason.

### Accord and discord with other studies

Our cohort size and observed relapse-free survival rates are comparable to those reported in other series on 2-stage revision arthroplasty patients. Sanchez-Sotelo et al. also found that uncemented stem fixation does not increase the risk for infection relapse after 2-stage revision arthroplasty [12]. Other studies demonstrated results similar to ours, following uncemented 2-stage revision even without the use of a spacer [13,14]. In a multi-center cohort of 221 patients, the relapse-free survival at 2 years was 94%; [15]. The fixation method was not examined in that study.

Russo et al. reported that cemented modular stems in 1-stage revisions yielded revision-free survival rates slightly superior to those for our cemented group, with comparable relapse-free survival [3]. The microbiological spectrum in that study was similar to that found in our cohort.

Outcomes for patients with uncemented stems in our study are comparable to those found in other cohort studies [16-18]; however, these prior studies did not include a control group.

Different failure modes have been observed and described across different fixation modes, with uncemented stems having more early failures, whereas cemented stems are more prone to late failure [19]. This is in agreement with our study and may explain our findings regarding better survival free from reoperation for any reason among patients operated on with uncemented stems.

Our findings indicate a lower incidence of *S. aureus* as well as methicillin-resistant *Staphylococcus aureus/epidermidis* (MRSA/MRSE) related PJI compared with data from European and American studies [20-23]. A possible explanation is that *S. aureus* infections have an obvious clinical presentation, and in our unit most patients affected by this agent were diagnosed early and treated with DAIR or 1-stage revision. Moreover, the studies already mentioned describe the microbiology of PJI in other countries. Sweden’s strict compliance with contemporary antibiotic guidelines has led to low levels of antibiotic resistance, consequently explaining the low prevalence of MRSA/MRSE [24].

A rising incidence of fungal PJIs is described internationally, posing a substantial therapeutic challenge [25,26]. Yet, a surprisingly low incidence of fungal pathogens (1 case of polymicrobial PJI) was observed within our cohort.

### Limitations

A major limitation of our study is the inherent selection bias. Stem selection is not arbitrary; it is determined by patient-specific factors that have the potential to directly influence the outcome. Older, osteoporotic patients and patients with large diaphyseal bone defects (Paprosky IIIB and IV) are more likely to undergo surgery with a cemented stem [19,27]. The younger mean age of patients with uncemented stems in our cohort corroborates this observation. Our retrospective study is also limited by unmeasured and unknown confounders. For example, information on body mass index, comorbidities, and an unhealthy lifestyle (e.g., smoking and alcohol use) is not included in this study.

As mentioned, our single-center cohort study derives from a local registry. Despite an estimated 100% completeness, the registry’s accuracy remains unverified. We reviewed the records of patients identified in the registry repeatedly to eliminate registration and transcription errors. The amount of missing data is thought to be minimal and unlikely to have influenced the events of the outcomes and the principal statistical analysis.

We have not cross-referenced our data with the Swedish Arthroplasty Register. However, as a referral hospital, we are regularly consulted regarding complications following surgeries on referred patients, particularly when these patients are in need of additional surgery, which is most often performed by our unit. Our qualified assumption is therefore that no events went unnoted, but this remains unverified.

We cannot completely ignore the estimated aHR for PJI relapse; at the same time, we observed inflated CIs and no statistical significance. The inflated CIs were expected given the relatively low number of included patients and the scarcity of events. Despite that, we identified a statistically significant difference in our secondary outcome. We are aware that the observed lack of a statistically significant difference in the risk of infection relapse between groups may be due to limited statistical power, opening up for type-2 errors. Moreover, given the observed CIs surrounding the estimate for the risk of infection relapse, the risk of this event could be either reduced or considerably higher after uncemented stem fixation. Our study therefore appears to be underpowered to evaluate associations of uncemented stem fixation with our primary outcome.

Given the inflated confidence intervals presented above, and given the risk of introducing over-adjustment, we refrained from including additional covariates into our regression models: We did not adjust for fixation method (cemented or uncemented) of the implants extracted at stage 1, i.e., prior to index surgery, which can affect 2-stage revision arthroplasty outcomes [14], because suboptimal removal of cement during stage 1 of a 2-stage revision could negatively affect the eradication of the PJI. We also did not adjust for the type of prosthetic joint (primary or secondary) revised prior to index surgery, but fewer secondary joints were present in the cemented group. The follow-up is statistically significantly shorter for patients with cemented stems. A transfemoral approach was more commonly used in the uncemented group compared with the cemented group, but this was also not adjusted for. These parameters, i.e., fewer secondary joints revised, shorter follow-up and less frequent use of transfemoral approaches in the cemented group, may have been favorable for the outcome in this group, which renders our findings on the slightly reduced risk of reoperation among patients receiving uncemented stems conservative.

We did not present data for antibiotic treatment. This data is of course part of the medical charts that we reviewed, and as a general principle the chosen antibiotic regimen was guided by the specific microbiological findings in each case and aligned with up-to-date guidelines. The antibiotic treatment was not influenced by the method of femoral stem fixation. Including information on the relatively wide variety of antibiotic regimes in the analysis would have introduced considerable statistical uncertainty.

### Strengths

Despite all the above limitations, this is only the second comparative study to address the association between the type of stem fixation in 2-stage hip revision for PJI and the risk of relapse, and the first to address reoperation for any reason [12]. Ours is also a large cohort compared with other studies on similar topics [11,13,14]. The size of our cohort was large enough to allow regression models to be fitted without violating model assumptions, and we limited the number of included covariates to avoid inflated CIs or over-adjustment. The covariates included in our models are considered the most relevant, but of course their choice can be questioned.

Furthermore, comprehensive microbiological data was available. Our study is believed to have relatively robust external validity at least within the setting of Nordic health care because our procedures and patient selection are fairly congruent with practice in these countries. This study focused on 2 key clinical parameters to define successful 2-stage revision arthroplasty: complete pathogen eradication, eliminating the need for further antibiotics, and the absence of any additional surgical intervention. In a discrete choice experiment, patients suffering from PJI considered “no need for further surgery” to be a highly prioritized outcome of their impending treatment [28]. While our study does not provide a conclusive answer regarding the risk of infection relapse after 2-stage revision arthroplasty for PJI, it does contribute meaningful evidence supporting the use of uncemented stem fixation in order to reduce the risk of any further surgery.

### Conclusion

We showed no statistical difference in risk of infection relapse but uncemented stem fixation in 2-stage revision arthroplasty for PJI was associated with a reduced risk of reoperation for any reason.

*In perspective,* the choice of femoral implant fixation in 2-stage revision arthroplasty of the hip could also be based on patient-related factors such as age, femoral bone loss, and general bone quality. The elevated risk of late complications associated with cemented stem fixation in 2-stage revision arthroplasty certainly warrants consideration of uncemented fixation, particularly in younger patients.

## Notes

GP: review of medical records, data collection, drafting and revision of the manuscript; AB: statistical analysis, review and editing of the manuscript, supervision; NPH study design, review and editing of the manuscript, supervision.

Handling co-editors: Marianne Westberg and Philippe Wagner

*Acta* thanks Carsten Frank Perka and Christian Thomas Pollmann for help with peer review of this manuscript.
